# Estimating Heart Failure Health Status From Multimodal Wearables: Implementation of a Feature-Engineering Framework for KCCQ-12 Prediction

**DOI:** 10.1016/j.mcpdig.2026.100403

**Published:** 2026-09-17

**Authors:** Christopher J. McLeod, Charles Bruce, Kuldeep Singh Rajput, Joseph S. Ross, Sy Jackie Lou Velarde, Felis Dwiyasa, Chen Hao, Yan He, Pyae Phyo Tun, Stephanie Zawada, Maulik Majmudar

**Affiliations:** a Department of Cardiovascular Diseases, Mayo Clinic, Jacksonville, FL; b OutcomesAI, Inc, formerly of Biofourmis, Inc, Singapore; c Section of General Internal Medicine, Yale School of Medicine, New Haven, CT; d Independent Researcher, Singapore, formerly of Biofourmis, Inc, Singapore; e Singapore Management University, Singapore, formerly of Biofourmis, Inc., Singapore; f Kern Center for the Science of Health Care Delivery, Mayo Clinic, Rochester, MN; g Biofourmis, Inc, Boston, MA

## Abstract

Routine monitoring of symptom burden and quality of life in heart failure (HF) patients is limited by intermittent sampling of validated instruments, such as the Kansas City Cardiomyopathy Questionnaire (KCCQ-12). As more wearable devices track physiological measures in real-world settings than ever before, we propose an exploratory implementation of a feature-engineering framework to bridge this sampling gap, approximating KCCQ-12 scores and patient health status from wearable features. In this narrative, we processed data from the 8-week multicenter observational study of patients with HF (New York Heart Association, class I-III) wearing Everion multisensor smartwatches: “Evaluating Mobile Health Tool Use for Capturing Patient-centered Outcomes Measures in Patients with HF” (NCT04191356). Then, Least Absolute Shrinkage and Selection Operator logistic regression was applied for feature selection, followed by binary classification and linear mixed-effects modeling to predict KCCQ-12 scores. Among patients generating sufficient data (n=59), there were 173 unique KCCQ-12 observations. From 7235 candidate features, Least Absolute Shrinkage and Selection Operator retained 88 predictors (Supplemental Table 1, available online at https://www.mcpdigitalhealth.org/), including the top predictors: passive functional capacity metrics (positive), daily steps/walking time (positive), nocturnal respiratory variability (negative), and patient-reported dyspnea (negative). Binary classification models achieved a test of area under the curve=0.77 (sensitivity, 91%; specificity, 63%), and linear mixed-effects models produced a root mean squared error of 14.7 (R^2^=0.58) for predicting patient health status and continuous KCCQ-12 scores, respectively. A patient-level split analysis revealed decreased model performance (root mean squared error=18.9; R^2^=0.33), suggesting limited generalizability to new individuals. This exploratory implementation with a small sample size outlines wearable features that warrant future investigation for KCCQ-12 score monitoring and offers early-stage lessons for assessing such features as proxies of KCCQ-12 scores in real-world settings.

The wave of successful remote monitoring (RM) pilot studies suggests that wearable technology, particularly tools that capture signals across multiple physiological and behavioral domains, can measure symptom changes in real-world settings.^1^, ^2^, ^3^, ^4^, ^5^ For patient populations whose disease management necessitates frequent sampling of biomarkers, such as those with heart failure (HF), measures captured in real-world settings can supplement current RM protocols that involve frequent sampling of lagging indicators, such as weight fluctuations.^6^ Although early research in the HF space focused on observational and interventional studies evaluating telehealth visits, remote coaching, and implantable devices, a growing number of RM studies use wearable devices with embedded sensors, like smartwatches, to capture data related to functional status, symptom burden, or quality of life in real-world settings.^7^, ^8^, ^9^, ^10^, ^11^

Among the most commonly used instruments to assess health status and hospitalization risk in patients with HF, the Kansas City Cardiomyopathy Questionnaire (KCCQ-12) is typically collected only during scheduled clinic visits, limiting its utility for continuous patient monitoring.^12^^,^^13^ In addition, the multidimensional nature of the KCCQ-12, spanning physical limitations, symptom burden, social limitations, and subjective patient perception, complicates its measurement outside clinical environments.^14^ Increasingly, the findings from wearable studies in HF populations suggest that multimodal data from wearables may facilitate frequent, reliable estimation of KCCQ-12 scores in real-world and community settings.^15^^,^^16^

Although previous studies have reported correlations and predictive value between individual wearable features (eg, activity levels, heart rate variability, etc) and clinical outcomes, less is known about strategies to identify those features useful for predicting patient-reported health status in HF populations. In this narrative, we discuss the implementation of an exploratory feature-engineering framework that uses longitudinal data from multimodal sensors embedded in a wearable to highlight digital features that warrant future investigation for KCCQ-12 score monitoring. This novel interpretable framework integrates wearable features from a set of domains broader than currently available research, spanning voice, heart rate, respiratory, and functional capacity features, to approximate patient health status and continuous KCCQ-12 scores, offering researchers a rationale for wearable features that may one day qualify as reliable proxies of KCCQ-12 scores in real-world settings.

### Implementation Data Sources and Characteristics

This exploratory implementation utilized data from an 8-week multicenter observational study (ClinicalTrials.gov NCT04191356) in which 65 patients with HF (New York Heart Association, class I-III) wore Everion multisensor smartwatches (Biovotion AG, Switzerland) continuously while completing biweekly KCCQ-12 assessments by a mobile application (BiovitalsHF).^17^ Eligible patients were adults (21-85 years) with documented HF and recent or current diuretic use, recruited from 6 institutions across the United States and Singapore. After informed consent was obtained from each participant, the wearable device captured accelerometry and photoplethysmography metrics. Patients also performed weekly voice recording tasks through smartphone. In addition, guided in-clinic 6-minute walk tests (6MWT) and home-based self-administered 6MWTs (using smartwatch accelerometry) were conducted biweekly. Extracted clinical data from electronic health records included demographics, HF etiology, comorbidities, medication regimens, and laboratory values (NT-proBNP).

Of the patients who enrolled in the study (n=65; mean age 56.7±11.7 years, 83% male; New York Heart Association, distribution I/II/III: 14/42/9), 59 provided sufficient data for analysis, yielding a total of 164 unique KCCQ-12 observations over the study period (median assessments per patient=3; IQR: 2-4). The median baseline KCCQ-12 overall summary score was 65 (IQR: 45-83), indicating moderate health status impairment.^12^

KCCQ-12 overall summary scores were dichotomized at the cohort baseline median (≥65 vs <65) for balanced classes in exploratory discrimination to predict patient health status. This is a sample-specific, data-driven cutoff, not a clinically anchored boundary: 65 falls within the conventional fair-to-good band (50-74) and should not be read as a recommended clinical decision threshold.

### Feature-Engineering Framework

#### Quality Control

Raw sensor streams from the smartwatch were transformed into clinically interpretable features, organized into hierarchical families (Supplemental Table 2, available online at https://www.mcpdigitalhealth.org/). Families were designed to capture distinct physiological and behavioral constructs relevant to health status. Raw wearable data underwent multistage quality control, including:

• Signal-quality filtering: each raw data stream was accompanied by a device-reported signal-quality score (0-100), generated by validated onboard algorithms that integrate signal stability, interbeat consistency, motion artifact burden, and sensor–skin contact quality. Data segments with scores <50 were excluded due to known degradation in signal reliability below this threshold.^18^ Quality control was applied at the segment level rather than the participant level, allowing retention of participants with sufficient high-quality data while ensuring robustness.
• Wear-time thresholds: only days with ≥8 hours of valid wear time were retained for daily aggregates, consistent with relevant literature.^19^
• Study artifact removal: actigraphy during scheduled clinic visits and supervised 6MWTs was excluded to ensure features reflected real-world behavior.
• Weekly aggregation requirements: weekly-level features required ≥3 valid days in the observation window.^20^

#### Feature Families

From quality-controlled sensor streams, 7235 candidate features were engineered across 8 feature families. Representative features are described in Table 1.

**Table 1:** Feature Categories with Number of Features, Rationale, and Representative Features

Domain	Features (N)	Rationale	Selected Representative Features
Passive functional capacity	143	Peak ambulatory capacity during daily life reflects functional reserve without clinic visit burden	Passive 6-minute walk distance (p6MWD, in meters); passive 6-minute step count (p6MW-steps); daily maximum sustained walk distance; percentiles (50th, 75th, and 95th) of 6-minute rolling window distances; weekly trends and variability in p6MWD; device-based (Everion vs Apple Watch) comparisons
Daily activity	892	Overall activity volume and patterns correlate with functional capacity and energy levels	Total daily steps (mean, median, SD, percentiles); daily walking time (minutes); sedentary time; time in moderate-to-vigorous physical activity; activity fragmentation (number of activity bouts, mean bout duration); circadian rhythm metrics (amplitude, acrophase, and mesor from cosinor analysis); day-to-day variability in steps; and weekend versus weekday patterns
Nocturnal respiratory	347	Breathing stability during sleep reflects congestion status, periodic breathing, and sleep-disordered breathing—key heart failure (HF) pathophysiology	Mean and SD of nocturnal respiratory rate; 5th, 50th, 95th percentiles; coefficient of variation; apnea-hypopnea index approximation; respiratory rate trend overnight (early vs late sleep); number of breathing irregularity episodes; and correlation between respiratory rate and heart rate during sleep
Heart rate and HRV	1456	Autonomic function, reflected by HRV, predicts HF outcomes; resting heart rate indicates hemodynamic compensation	Resting heart rate (mean, nighttime minimum, and morning average); time-domain HRV (SDNN and RMSSD); frequency-domain HRV (LF, HF, and LF/HF ratio); heart rate recovery after activity bouts; heart rate response to postural changes; and diurnal heart rate variation
Oxygen saturation	284	SpO_2_ levels reflect pulmonary function and perfusion adequacy, particularly relevant in HF with pulmonary congestion	Mean and minimum SpO_2_ (overall, nocturnal, and during activity); desaturation episodes (frequency, duration, and nadir); variability of SpO_2_; time <90% saturation threshold
Acoustic/voice biomarkers	3524	Voice characteristics (breathiness, fundamental frequency variability, and pause patterns) may reflect dyspnea, fluid status, or fatigue	Mel-frequency cepstral coefficients; fundamental frequency (F0) statistics; jitter and shimmer (voice stability); pause duration (mean, max, frequency); speech rate; spectral centroid, roll-off, and flux; zero-crossing rate; energy and loudness contours
Clinical and demographic characteristic variables	412	Baseline clinical characteristics and treatments influence health status independently of sensor data	Age, sex, BMI, HF etiology, NYHA class, and ejection fraction category; comorbidities (diabetes, atrial fibrillation, CKD, and COPD); medication classes (ACE-I/ARB, β-blockers, MRA, SGLT2i, and diuretic dose); baseline NT-proBNP; and previous hospitalization count
Patient-reported symptoms	177	Daily symptom reports provide complementary subjective data about dyspnea, fatigue, and edema	Dyspnea severity score (0-10 scale); fatigue level; peripheral edema presence; symptom frequency; symptom trend (improving, stable, and worsening); correlation between symptoms and activity levels

Abbreviations: ACE-I, ACE inhibitors; ARB, angiotensin receptor blockers; CKD, chronic kidney disease; COPD, chronic obstructive pulmonary disease; HF, high frequency; HRV, heart rate variability; LF, low frequency; MRA, mineralocorticoid receptor antagonists; NT-proBNP, N-terminal pro-b-type natriuretic peptide; NYHA, New York Heart Association; RMSSD, root mean square of successive differences; SDNN, standard deviation of normal-to-normal intervals; SGLT2i, SGLT2 inhibitor; SpO2, peripheral capillary oxygen saturation.

#### Temporal Alignment and Aggregation

For each KCCQ-12 assessment timepoint (observation), we extracted features from the preceding 7-day observation window (or 14 days for biweekly assessments), ensuring temporal precedence for prediction.^21^^,^^22^ Features were aggregated using statistical summaries (mean, median, SD, min, max, and percentiles), change metrics (slopes, differences from baseline), and variability measures. This yielded the final 7235-dimensional feature space per KCCQ-12 observation.

Missing data were handled through multiple imputation for clinical variables (5 imputations using chained equations) and forward filling for sensor metrics when <2 consecutive days were missing. Only observations with <30% missing features were included.^23^^,^^24^ These procedures assume missingness is at least conditionally at random in this exploratory implementation.

#### LASSO Regularization for Feature Selection

Given the high-dimensional feature space (*P*=7.235) relative to sample size (n=164 analyzable observations), the Least Absolute Shrinkage and Selection Operator (LASSO) regression was used to identify the most informative predictors while preventing overfitting.^25^^,^^26^ LASSO applies L1 regularization, shrinking coefficients of uninformative features to exactly 0, thereby performing automatic feature selection.^27^

Features were standardized (z-score normalization) before LASSO to ensure equal penalization regardless of measurement scale. The regularization parameter (λ) was optimized by 5-fold cross-validation on the training set, selecting the λ value that minimized classification error (for logistic LASSO) or mean squared error (for linear LASSO). The 1-standard-error rule was applied to favor sparser models within 1 SE of the minimum error.^28^

#### Training, Validation, and Testing Strategy

The framework (Figure) deployed and model developed in this implementation took shape after inspecting the data. Time-based data splitting was applied to simulate prospective deployment: observations from the first 70% of study duration formed the training set (n=115), and the final 30% formed the test set (n=49). This approach maintains temporal ordering while testing model prediction of future assessments. Within the training set, 5-fold cross-validation optimized hyperparameters (λ for LASSO; random effects structure for linear mixed-effects models). Final models were trained on the full training set with optimized parameters and then evaluated on the test set. Patient-level split analysis was also performed: training on 70% of patients and testing on the remaining 30%.

**Figure:**
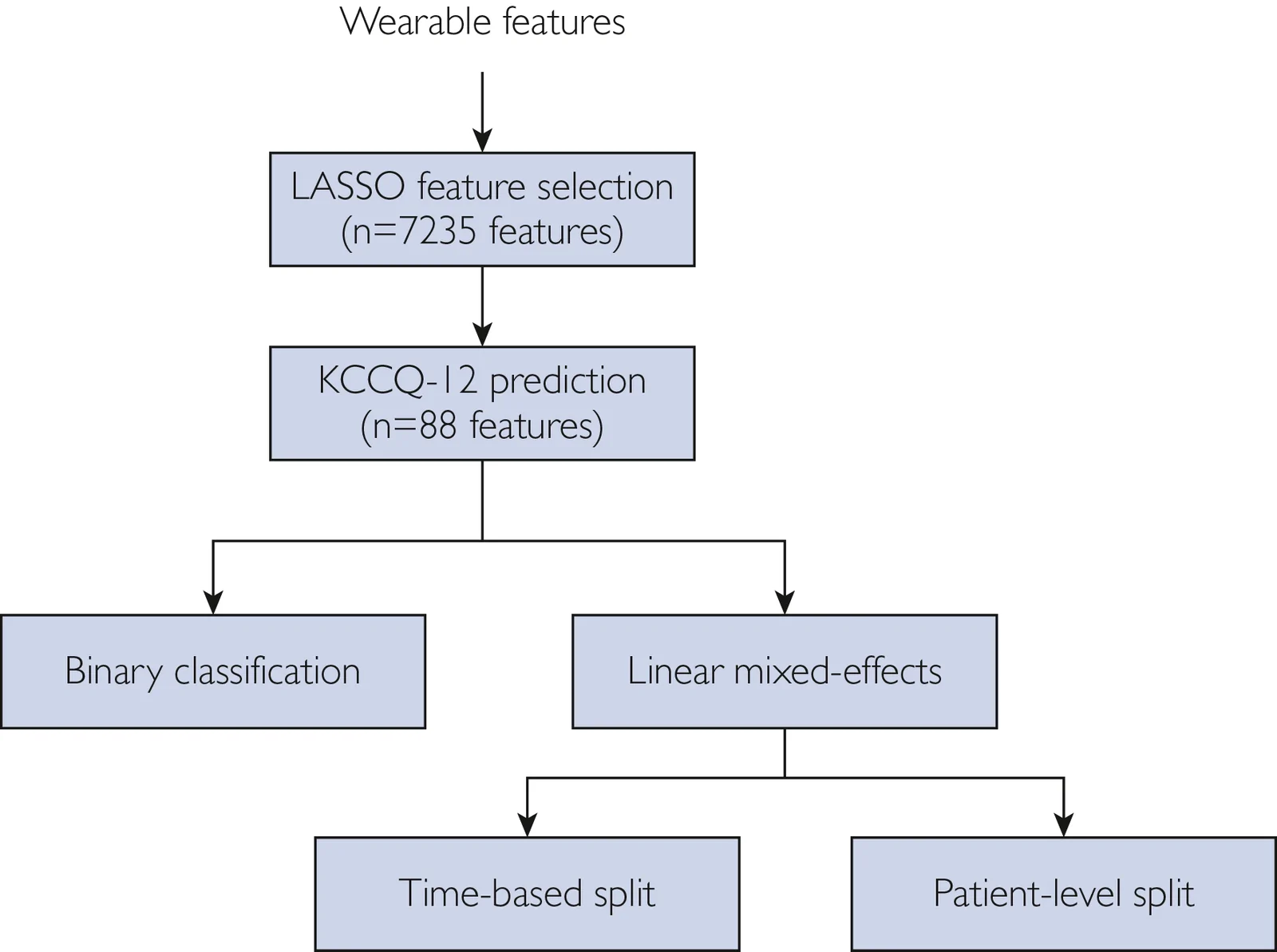
Feature-engineering framework.

To understand which features, contribute most to predicting KCCQ-12 scores, feature importance from LASSO coefficients (standardized β weights) was ranked. For linear mixed-effects models, fixed effects coefficients were reported with 95% confidence intervals and *P* values. All data processing and feature selection were performed on the training data exclusively.

The time-based split retains each patient’s earlier observations in the training set and later observations in the test set. It estimates within-patient prediction at a future timepoint in patients already represented in training, not generalization to new individuals. The patient-level split, with all observations from a patient confined to one side of the partition, provides that estimate and is reported as our primary measure of generalization.

### Feature Selection and Top Predictors

LASSO regularization retained 88 features from the original 7235, reducing dimensionality by 98.8%, spanning all 8 feature families.^29^ These features spanned all included domains: passive functional capacity (n=12 features), daily activity (n=23), nocturnal respiratory (n=15), heart rate/heart rate variability (n=14 features), SpO_2_ (n=8), voice biomarkers (n=7), clinical variables (n=6), and patient symptoms (n=3). Features with the strongest association with KCCQ-12 score are described in Table 2 (Supplemental Figure 1, available online at https://www.mcpdigitalhealth.org/).

**Table 2:** Top 10 Features Ranked by Absolute LASSO Coefficient Magnitude (From Strongest to Weakest Association With KCCQ-12)

Domain	Feature	LASSO Coefficient Magnitude	Interpretation
Passive functional capacity	Passive 6-minute walk distance (p6MWD)	β=+1.84	Higher p6MWD strongly associated with better KCCQ-12
Nocturnal respiratory	SD of nocturnal respiratory rate	β=−1.67	Greater breathing irregularity during sleep associated with worse health status
Daily activity	Daily step count	β=+1.52	Higher daily activity volume predicts better KCCQ
Patient-reported symptoms	Patient-reported dyspnea severity	β=−1.44	Higher subjective breathlessness predicts lower KCCQ
Daily activity	Daily walking time	β=+1.28	More time spent walking associated with better status
Nocturnal respiratory	95th percentile nocturnal respiratory rate	β=−1.19	Elevated peak nighttime breathing rate predicts worse KCCQ
Acoustic/voice biomarkers	Morning heart rate variability (SDNN)	β=+1.06	Better autonomic function associated with better health status
Nocturnal respiratory	Mean nocturnal SpO_2_	β=+0.98	Higher overnight oxygen saturation predicts better KCCQ
Acoustic/voice biomarkers	Voice pause duration	β=−0.91	Longer pauses during speech (suggesting dyspnea/fatigue) predict lower KCCQ
Clinical and demographic characteristics variables	β-blocker use	β=+0.87	Patients on β-blockers reported slightly better health status after covariate adjustment

KCCQ, Kansas City Cardiomyopathy Questionnaire; SDNN, standard deviation of normal-to-normal intervals; SpO2, peripheral capillary oxygen saturation.

### Model Performance

The test-set mean absolute error (11.2 points) and root mean squared error (RMSE, 14.7 points) meet the clinically meaningful KCCQ-12 change threshold (∼5-10 points). In the patient-level split analysis (training on 41 patients, testing on 18 patients), model performance decreased (test R^2^=0.33, RMSE=18.9 points), indicating limited generalization of the model to new individuals (Table 3). The high ratio of predictors to observations likely contributes to this decrease.

**Table 3:** Model Performance Specifications

Model Type	Training Set Performance	Test Set Performance (Supplemental Figure 2)
Binary classification	n=115, 5-fold CV; AUC=0.91 (95% CI, 0.86-0.96); sensitivity=97%, specificity=81%; and F1 score=0.89	n=49, held-out; AUC=0.77 (95% CI, 0.64-0.90); sensitivity=91%, specificity=63%; and F1 score=0.74
Continuous score linear mixed-effects model	R^2^=0.86 (marginal R^2^=0.71 for fixed effects alone); RMSE=8.3 points, MAE=6.4 points; Pearson r=0.93 (predicted vs actual)	R^2^=0.58 (marginal R^2^=0.44); RMSE=14.7 points, MAE=11.2 points; and Pearson r=0.77 (predicted vs actual)

AUC, area under the curve; CI, confidence interval; MAE, mean absolute error; RMSE, root mean squared error.

### Lessons Learned for Future Research

This implementation describes a novel feature-engineering framework, spanning wearable features from actigraphy, physiology, acoustic, and clinical domains, that identifies interpretable and clinically meaningful predictors of patient-reported health status in a small HF cohort. From 7235 candidate features spanning actigraphy, physiology, acoustic, and clinical domains, regularized modeling identified 88 key predictors that collectively achieved good discrimination of health status (area under the curve=0.77) and explained 58% of the variance in continuous KCCQ-12 scores (Supplemental Figure 2, available online at https://www.mcpdigitalhealth.org/). Importantly, the retained features exhibited strong physiological coherence, clustering around functional capacity, respiratory stability, autonomic tone, oxygenation, and symptom burden.^7^^,^^10^ The combination of passive functional capacity metrics (passive 6-minute walk distance) with nocturnal respiratory monitoring represents a novel feature pairing that warrants future investigation beyond an implementation in observational and randomized controlled studies.

The top predictive features report physiological coherence: passive functional capacity (passive 6-minute walk distance, daily steps/walking time) emerged as the strongest positive predictors, while nocturnal respiratory instability (breathing rate variability and elevated peak rates) and symptom burden (dyspnea severity and voice changes) were the strongest negative predictors. Combined, these features (exercise capacity, congestion status, and subjective symptom experience) might offer multidimensional insights into the relationship between HF physiology and patient-reported health status. The LASSO regularization framework provides automatic feature selection while maintaining interpretability, whereas the ranking of features by coefficient magnitude provides immediate insight into relative importance. Although the features identified as predictive of KCCQ-12 are physiologically plausible, LASSO-selected coefficients can be unstable in small cohorts and scenarios in which features are highly correlated. Additionally, cross-validation folds used during feature selection could not be verified as patient-grouped, and grouped or nested cross-validation could not be performed retrospectively in this implementation. Cross-validated performance may be optimistic; the patient-level held-out evaluation is the more reliable estimate of generalization to new patients. Furthermore, the use of mixed-effects modeling with patient random intercepts acknowledges and quantifies between-patient heterogeneity while leveraging within-patient repeated measures.

A key contribution of this implementation to the literature is the transparent feature-engineering approach. Each of the 7235 candidate features were engineered with explicit physiological rationale, organized into interpretable families (functional capacity, daily activity, etc.), and derived using documented aggregation rules. This contrasts with “black box” deep learning approaches that operate on raw sensor streams without interpretable intermediate representations, offering future researchers a rationale for exploring these predictors and HF patient-reported outcomes.

The modest sample size (n=59) limited statistical power, particularly for patient-level generalization analyses, and the short study duration limits assessment of long-term prediction accuracy. The predominantly male and relatively young cohort limited generalizability to older, frailer patients. Another critical limitation of this approach was that model calibration was not assessed: calibration curves, calibration intercept and slope, Brier score, and prediction-interval coverage were not evaluated, and calibration of these models is unestablished. Good discrimination can coexist with poor calibration, making this a material invitation for individual-level application. Formal calibration assessment is a pre-specific objective of our future validation study in a larger, independent cohort. Another limitation is that the binary threshold for the cohort was defined by its median. Alternative clinically anchored cutoffs, such as 50 or 75, were not evaluated, so the robustness of classification performance to threshold selection is unknown. Future researchers should address calibration of models and sensitivity analyses assessing clinically relevant cutoffs for patient-reported outcomes.

Missingness in passively collected data was likely informative in this cohort. Mean device wear was 13.7 hours per day among completers vs 3.5 hours per day among those who withdrew (at least 8 hours of daily wear: 88.5% vs 21.4%), and questionnaire completion was 95.4% vs 60.4%, suggesting that reduced engagement co-occurred with a poorer clinical trajectory. Signal-quality filtering may compound this bias if poor signal quality coincides with illness or inactivity. Forward-filling and complete-case approaches carry opposing biases; however, we did not perform sensitivity analyses using complete-case data, unfilled features, stricter wear-time thresholds, or explicit missingness indicators. Estimates may be biased toward periods of better health, and the magnitude of that bias is unquantified. Future investigators should consider these limitations and biases when designing hypotheses in this research area.

### Future Directions

Beyond this exploratory implementation, future studies evaluating this exploratory implementation must prioritize multisite external validation with patient populations across age groups, sex, race, HF phenotypes.^30^^,^^31^ Longer studies (>6 months) are needed to assess longitudinal tracking of disease progression. Prospective validation comparing model predictions to subsequent clinical events, such as hospitalizations, would establish clinical utility beyond KCCQ estimation. As the cutoff KCCQ-12 score used in this implementation emerged from this specific dataset, future models should perform appropriate sensitivity analyses or use other specified cutoffs to assess model performance.

The model’s 88-feature structure remains complex for manual clinical application. Future researchers should prioritize the inclusion of predictors of known physiological importance or combinations of predictors that significantly improve model performance. As the model explained, only 58% of KCCQ-12 variance, the incorporation of additional data sources, such as electronic health record, imaging, or blood work data, could improve prediction. Alternatively, comparison to ensemble methods, such as random forests, and temporal models, such as recurrent neural networks, could yield optimized models. Methods to leverage initial patient data for individual baseline calibration might also improve new-patient generalization.

## Conclusion

In this narrative, we outline a feature-engineered framework for estimating patient-reported health status in HF using multimodal wearable features. Using a systematic approach to feature selection with LASSO regression, the final binary classification model predicted KCCQ-12 scores (area under the curve=0.77 for binary classification of patient health status; R^2^=0.58 and RMSE of 14.7 for continuous prediction); however, the generalizability of our approach remains limited, given that a patient-level split analysis revealed decreased model performance (RMSE=18.9; R^2^=0.33). This implementation revealed that top predictors (passive functional capacity, nocturnal breathing stability, daily activity levels, and symptom burden) aligned with established HF pathophysiology. This initial implementation of our novel framework offers early-stage evidence for future validation and randomized controlled studies using wearable features to predict HF patient-reported outcomes. As RM becomes standard in HF management, improved feature-engineering approaches like this will be essential for translating wearable features captured in real-world settings into actionable clinical insights.

## Potential of Competing Interests

This publication was supported by the Food and Drug Administration (FDA) of the US Department of Health and Human Services (HHS) as part of a financial assistance award (Center of Excellence in Regulatory Science and Innovation grant to Yale University and Mayo Clinic, U01FD005938) totaling US $308,129, with 100% funded by FDA/HHS and in-kind support from Biofourmis. The contents are those of the authors and do not necessarily represent the official views of nor an endorsement by FDA/HHS or the United States Government. Dr Ross currently receives research support through Yale University from Johnson and Johnson to develop methods of clinical trial data sharing, from the Food and Drug Administration for the Yale-Mayo Clinic Center for Excellence in Regulatory Science and Innovation program (U01FD005938), from the National Institutes of Health (R01AA032254), from the Greenwall Foundation, and from Arnold Ventures. No other authors have conflicts to disclose.

## Ethics Statement

This study was conducted in compliance with the Declaration of Helsinki regarding ethics for medical research involving human subjects. Patients were informed of their right to object to the use of their data for the present study and gave informed consent.
