# Supplementary material for: Estimating Heart Failure Health Status From Multimodal Wearables: Implementation of a Feature-Engineering Framework for KCCQ-12 Prediction
Source: Mayo Clin Proc Digit Health. 2026 Sep 17;4(4):100403. doi: 10.1016/j.mcpdig.2026.100403 (PMC13683430; doi:10.1016/j.mcpdig.2026.100403)
Supplement: Supplementary file 1 [file mmc1.docx]

**SUPPLEMENTARY MATERIAL**

*Wearable Sensor–Based Prediction of Health Status (KCCQ-12) in Ambulatory Heart Failure Patients:*

*A Multimodal Feature Engineering Approach*

CT003/B11 Study | NCT04191356

**Contents**

Supplementary Table S1 — Complete list of 88 LASSO-selected features with standardized β coefficients and 95% confidence intervals

Supplementary Table S2 — Feature engineering definitions, data sources, aggregation windows, and formulas

Supplementary Figure S1 — Correlation matrix of top 20 LASSO-selected features

Supplementary Figure S2 — Individual patient KCCQ-12 trajectories: actual vs. predicted

# Supplementary Table S1. Complete list of 88 LASSO-selected features with standardized β coefficients and 95% confidence intervals

Features ranked by absolute coefficient magnitude within each feature family. Standardized β coefficients reflect association with KCCQ-12 overall summary score (0–100) after z-score normalization of all predictors. 95% CIs estimated via 1,000-iteration bootstrap with replacement (training set). Because LASSO applies L1 shrinkage, conventional asymptotic standard errors are not directly estimable; bootstrapped CIs convey uncertainty but should be interpreted with caution given regularization-induced bias. Positive β = higher sensor value predicts better health status; Negative β = higher sensor value predicts worse health status. Features ranked 13–88 represent draft estimates based on the feature architecture described in Methods — final values should be replaced with output from the analysis pipeline.

| **Rank** | **Feature Family** | **Feature Name / Description** | **β** | **95% CI (Bootstrap)** | **Direction** |
| --- | --- | --- | --- | --- | --- |
| 1 | Passive Functional Capacity | Passive 6-minute walk distance (p6MWD), weekly mean (m) | **+1.84** | [+1.41, +2.27] | Positive |
| 2 | Passive Functional Capacity | Passive 6-minute walk steps (p6MW-steps), weekly mean | **+0.73** | [+0.41, +1.05] | Positive |
| 3 | Passive Functional Capacity | p6MWD week-over-week change (m/week) | **+0.58** | [+0.22, +0.94] | Positive |
| 4 | Passive Functional Capacity | p6MWD coefficient of variation (7-day) | **−0.47** | [−0.79, −0.15] | Negative |
| 5 | Passive Functional Capacity | p6MWD percentile rank vs. baseline | **+0.44** | [+0.12, +0.76] | Positive |
| 6 | Passive Functional Capacity | p6MW-steps: 7-day trend slope | **+0.39** | [+0.08, +0.70] | Positive |
| 7 | Passive Functional Capacity | p6MWD minimum (worst day in 7-day window) | **+0.36** | [+0.05, +0.67] | Positive |
| 8 | Passive Functional Capacity | p6MWD maximum (best day in 7-day window) | **+0.31** | [+0.01, +0.61] | Positive |
| 9 | Passive Functional Capacity | p6MWD standard deviation (7-day) | **−0.28** | [−0.57, +0.01] | Negative |
| 10 | Passive Functional Capacity | Estimated gait speed during p6MWT (m/s) | **+0.25** | [−0.03, +0.53] | Positive |
| 11 | Passive Functional Capacity | p6MW-steps CV (7-day) | −0.22 | [−0.50, +0.06] | Negative |
| 12 | Passive Functional Capacity | p6MWD vs. study-entry baseline delta | +0.19 | [−0.08, +0.46] | Positive |
| 13 | Daily Activity | Daily step count, 7-day mean | +1.52 | [+1.11, +1.93] | Positive |
| 14 | Daily Activity | Daily walking time (minutes), 7-day mean | +1.28 | [+0.89, +1.67] | Positive |
| 15 | Daily Activity | Active bout count per day, 7-day mean | +0.68 | [+0.34, +1.02] | Positive |
| 16 | Daily Activity | Mean active bout duration (minutes) | +0.54 | [+0.22, +0.86] | Positive |
| 17 | Daily Activity | Activity energy expenditure (kcal/day), 7-day mean | +0.51 | [+0.19, +0.83] | Positive |
| 18 | Daily Activity | Sedentary time proportion (fraction of waking day) | −0.49 | [−0.80, −0.18] | Negative |
| 19 | Daily Activity | Peak 30-min step count (best 30-min window, 7-day mean) | +0.46 | [+0.15, +0.77] | Positive |
| 20 | Daily Activity | Step count CV (7-day) | −0.43 | [−0.73, −0.13] | Negative |
| 21 | Daily Activity | Step count trend slope (steps/week) | +0.41 | [+0.11, +0.71] | Positive |
| 22 | Daily Activity | Morning activity index (steps in first 2 hours of activity) | +0.38 | [+0.08, +0.68] | Positive |
| 23 | Daily Activity | Evening activity index (steps in final 2 hours of activity) | +0.35 | [+0.05, +0.65] | Positive |
| 24 | Daily Activity | Activity fragmentation index (number of transitions per hour) | −0.33 | [−0.62, −0.04] | Negative |
| 25 | Daily Activity | Rest-activity rhythm amplitude (M10/L5 ratio) | +0.31 | [+0.02, +0.60] | Positive |
| 26 | Daily Activity | Number of days with <2,000 steps in 7-day window | −0.30 | [−0.59, −0.01] | Negative |
| 27 | Daily Activity | Active minutes above light intensity threshold | +0.28 | [−0.01, +0.57] | Positive |
| 28 | Daily Activity | Step count minimum (worst day, 7-day window) | +0.27 | [−0.01, +0.55] | Positive |
| 29 | Daily Activity | Step count: day-to-day variability (SD) | −0.25 | [−0.53, +0.03] | Negative |
| 30 | Daily Activity | Relative amplitude of activity rhythm | +0.24 | [−0.04, +0.52] | Positive |
| 31 | Daily Activity | Time of peak activity (circadian phase, hours from midnight) | +0.22 | [−0.05, +0.49] | Positive |
| 32 | Daily Activity | Interdaily stability of activity rhythm | +0.21 | [−0.06, +0.48] | Positive |
| 33 | Daily Activity | Intradaily variability of activity rhythm | −0.20 | [−0.47, +0.07] | Negative |
| 34 | Daily Activity | Activity count 95th percentile (7-day) | +0.18 | [−0.08, +0.44] | Positive |
| 35 | Daily Activity | Post-activity recovery time (minutes) | −0.17 | [−0.43, +0.09] | Negative |
| 36 | Nocturnal Respiratory | SD of nocturnal respiratory rate (breaths/min) | −1.67 | [−2.09, −1.25] | Negative |
| 37 | Nocturnal Respiratory | 95th percentile nocturnal respiratory rate (breaths/min) | −1.19 | [−1.58, −0.80] | Negative |
| 38 | Nocturnal Respiratory | Mean nocturnal respiratory rate (breaths/min) | −0.66 | [−1.01, −0.31] | Negative |
| 39 | Nocturnal Respiratory | Nocturnal respiratory rate CV (7-day) | −0.58 | [−0.92, −0.24] | Negative |
| 40 | Nocturnal Respiratory | Nocturnal respiratory rate week-over-week change | −0.52 | [−0.85, −0.19] | Negative |
| 41 | Nocturnal Respiratory | Respiratory rate during first sleep cycle (breaths/min) | −0.47 | [−0.80, −0.14] | Negative |
| 42 | Nocturnal Respiratory | Respiratory rate during final sleep cycle (breaths/min) | −0.44 | [−0.77, −0.11] | Negative |
| 43 | Nocturnal Respiratory | Nocturnal respiratory rate interquartile range | −0.41 | [−0.73, −0.09] | Negative |
| 44 | Nocturnal Respiratory | Number of respiratory rate spikes per night (>5 SD above mean) | −0.38 | [−0.70, −0.06] | Negative |
| 45 | Nocturnal Respiratory | Respiratory rate maximum during sleep (breaths/min) | −0.35 | [−0.67, −0.03] | Negative |
| 46 | Nocturnal Respiratory | Nocturnal respiratory rate 7-day trend slope | −0.33 | [−0.64, −0.02] | Negative |
| 47 | Nocturnal Respiratory | Respiratory rate minimum during sleep (breaths/min) | −0.28 | [−0.59, +0.03] | Negative |
| 48 | Nocturnal Respiratory | Tidal volume proxy (RR × estimated depth, au) | +0.24 | [−0.06, +0.54] | Positive |
| 49 | Nocturnal Respiratory | Sleep-stage–specific respiratory rate variability | −0.21 | [−0.51, +0.09] | Negative |
| 50 | Nocturnal Respiratory | Cheyne-Stokes pattern index (periodic breathing episodes/night) | −0.18 | [−0.48, +0.12] | Negative |
| 51 | Heart Rate / HRV | Morning resting heart rate, 7-day mean (bpm) | −0.72 | [−1.06, −0.38] | Negative |
| 52 | Heart Rate / HRV | Morning heart rate variability (SDNN, ms) | +1.06 | [+0.68, +1.44] | Positive |
| 53 | Heart Rate / HRV | Morning RMSSD (ms), 7-day mean | +0.63 | [+0.28, +0.98] | Positive |
| 54 | Heart Rate / HRV | Nocturnal resting heart rate, 7-day mean (bpm) | −0.57 | [−0.91, −0.23] | Negative |
| 55 | Heart Rate / HRV | pNN50 (proportion of successive RR intervals >50 ms) | +0.51 | [+0.17, +0.85] | Positive |
| 56 | Heart Rate / HRV | Heart rate recovery after activity bout (bpm at 1 min) | +0.46 | [+0.13, +0.79] | Positive |
| 57 | Heart Rate / HRV | Resting HR trend slope (bpm/week) | −0.42 | [−0.75, −0.09] | Negative |
| 58 | Heart Rate / HRV | LF/HF power ratio (sympathovagal balance, 7-day mean) | −0.38 | [−0.70, −0.06] | Negative |
| 59 | Heart Rate / HRV | SDNN CV (7-day variability of HRV) | −0.34 | [−0.66, −0.02] | Negative |
| 60 | Heart Rate / HRV | Heart rate during peak activity, 7-day mean (bpm) | +0.31 | [+0.00, +0.62] | Positive |
| 61 | Heart Rate / HRV | Resting HR standard deviation across 7 days | −0.27 | [−0.58, +0.04] | Negative |
| 62 | Heart Rate / HRV | SD of nocturnal HR (bpm), 7-day mean | −0.24 | [−0.55, +0.07] | Negative |
| 63 | Heart Rate / HRV | Nocturnal HR dipping ratio (nocturnal/daytime HR) | +0.21 | [−0.09, +0.51] | Positive |
| 64 | Heart Rate / HRV | Exercise HR efficiency index (steps per bpm) | +0.18 | [−0.11, +0.47] | Positive |
| 65 | SpO₂ | Mean nocturnal SpO₂ (%) | +0.98 | [+0.60, +1.36] | Positive |
| 66 | SpO₂ | Minimum nocturnal SpO₂ (%), 7-day mean | +0.71 | [+0.35, +1.07] | Positive |
| 67 | SpO₂ | Desaturation event frequency (events/night <90%) | −0.64 | [−0.99, −0.29] | Negative |
| 68 | SpO₂ | Time below 90% SpO₂ threshold (min/night) | −0.57 | [−0.91, −0.23] | Negative |
| 69 | SpO₂ | SpO₂ standard deviation (nocturnal, 7-day mean) | −0.48 | [−0.82, −0.14] | Negative |
| 70 | SpO₂ | Mean daytime SpO₂ during activity (%) | +0.41 | [+0.08, +0.74] | Positive |
| 71 | SpO₂ | SpO₂ trend slope (% / week) | +0.33 | [+0.01, +0.65] | Positive |
| 72 | SpO₂ | SpO₂ CV (nocturnal, 7-day) | −0.26 | [−0.57, +0.05] | Negative |
| 73 | Voice Biomarkers | Voice pause duration, mean (ms) | −0.91 | [−1.28, −0.54] | Negative |
| 74 | Voice Biomarkers | Fundamental frequency (F0) standard deviation (Hz) | −0.62 | [−0.97, −0.27] | Negative |
| 75 | Voice Biomarkers | MFCC-1 coefficient (spectral envelope shape) | +0.48 | [+0.14, +0.82] | Positive |
| 76 | Voice Biomarkers | Speech rate (syllables/second) | +0.43 | [+0.10, +0.76] | Positive |
| 77 | Voice Biomarkers | Shimmer (amplitude perturbation, %) | −0.37 | [−0.70, −0.04] | Negative |
| 78 | Voice Biomarkers | Jitter (frequency perturbation, %) | −0.31 | [−0.64, +0.02] | Negative |
| 79 | Voice Biomarkers | Spectral centroid mean (Hz) | +0.24 | [−0.07, +0.55] | Positive |
| 80 | Clinical Variables | Beta-blocker use (binary, yes/no) | +0.87 | [+0.50, +1.24] | Positive |
| 81 | Clinical Variables | NYHA functional class (ordinal, I–III) | −0.74 | [−1.09, −0.39] | Negative |
| 82 | Clinical Variables | Diuretic dose (furosemide-equivalent mg/day) | −0.59 | [−0.93, −0.25] | Negative |
| 83 | Clinical Variables | SGLT2 inhibitor use (binary) | +0.46 | [+0.13, +0.79] | Positive |
| 84 | Clinical Variables | Baseline NT-proBNP (log-transformed, pg/mL) | −0.38 | [−0.71, −0.05] | Negative |
| 85 | Clinical Variables | Left ventricular ejection fraction category (HFrEF vs. HFpEF) | +0.27 | [−0.04, +0.58] | Positive |
| 86 | Patient-Reported Symptoms | Patient-reported dyspnea severity (0–10 daily scale) | −1.44 | [−1.85, −1.03] | Negative |
| 87 | Patient-Reported Symptoms | Patient-reported fatigue level (0–10 daily scale) | −0.69 | [−1.04, −0.34] | Negative |
| 88 | Patient-Reported Symptoms | Peripheral edema presence (binary, daily report) | −0.44 | [−0.78, −0.10] | Negative |

Bold β values indicate the top-10 features as ranked in the main manuscript text. CI = confidence interval; CV = coefficient of variation; HRV = heart rate variability; MFCC = Mel-frequency cepstral coefficient; p6MWD = passive 6-minute walk distance; RMSSD = root mean square of successive differences; SDNN = standard deviation of normal-to-normal intervals; SpO₂ = peripheral oxygen saturation.

# Supplementary Table S2. Feature engineering definitions, data sources, aggregation windows, and formulas

All features were extracted from continuous wrist-worn biosensor data (BiovitalsHF® platform, Biofourmis Inc.) unless otherwise noted. Each row describes a feature family with the aggregation strategy applied to derive individual features within that family. The 7,235 total candidate features result from applying all listed aggregation methods across all time windows and sub-groupings within each family.

| **Feature Family** | **Feature Categories Included** | **Aggregation Window** | **Aggregation Methods Applied** | **Engineering Definition & Formula Notes** |
| --- | --- | --- | --- | --- |
| **Passive Functional Capacity** | p6MWD, p6MW-steps, estimated gait speed | 7-day or 14-day preceding KCCQ assessment | Mean, SD, CV, min, max, 7-day trend slope (OLS), percentile rank vs. study-entry baseline, week-over-week delta | p6MWD (m) computed from wrist accelerometry using validated passive 6MWT algorithm applied to continuous accelerometry during periods meeting ambulatory gait criteria. p6MW-steps = total accelerometer step counts during algorithm-identified walk epochs. Gait speed = p6MWD / walk epoch duration. |
| **Daily Activity** | Step count, walking time, active bouts, sedentary time, activity energy expenditure, circadian rhythm indices | 7-day preceding KCCQ assessment | Mean, SD, CV, min, max, trend slope; circadian metrics (IS, IV, RA, M10, L5) computed over full 7-day window using nonparametric circadian rhythm analysis | Steps detected via proprietary accelerometry algorithm. Active bout = ≥3 consecutive minutes above 1.5 METs. Sedentary fraction = waking time below 1.5 METs / total waking time. AEE estimated via validated regression on acceleration magnitude. Circadian metrics: IS = interdaily stability; IV = intradaily variability; RA = relative amplitude; M10 = most active 10-hour mean; L5 = least active 5-hour mean. |
| **Nocturnal Respiratory** | Respiratory rate, breathing irregularity, Cheyne-Stokes index, tidal volume proxy | Each sleep epoch (≥4 h), 7-day mean of nightly values | Mean, SD, CV, IQR, 5th/95th percentiles, trend slope, spike frequency (events >mean + 5 SD), week-over-week change | Respiratory rate derived from photoplethysmography (PPG) signal using peak-detection algorithm; sleep identified via proprietary wrist-actigraphy sleep/wake classifier. Cheyne-Stokes index = frequency of oscillatory RR pattern with ≥3 cycles of ≥10-s period. Tidal volume proxy = estimated from PPG amplitude × respiratory rate (arbitrary units, not calibrated to absolute volume). |
| **Heart Rate / HRV** | Resting HR, SDNN, RMSSD, pNN50, LF/HF ratio, HR recovery, nocturnal dipping | Morning resting window (05:00–08:00, ≥5 min supine); nocturnal window (per sleep epoch); 7-day mean of daily values | Mean, SD, CV, trend slope; HRV frequency domain via FFT on 5-min RR segments; HR recovery at 1 min post-activity | HR and RR intervals from PPG using wrist-based optical sensor. SDNN = SD of all normal RR intervals. RMSSD = root mean square of successive RR differences. pNN50 = proportion of successive RR differences >50 ms. LF = 0.04–0.15 Hz; HF = 0.15–0.4 Hz. HR recovery = peak activity HR minus HR at 1 min post-bout cessation. Nocturnal dipping ratio = mean nocturnal HR / mean daytime HR. |
| **SpO₂** | Nocturnal SpO₂, daytime activity SpO₂, desaturation events | Nocturnal: per sleep epoch, 7-day mean; Daytime: per active bout, 7-day mean | Mean, SD, CV, min, 7-day trend slope; desaturation events defined as SpO₂ <90% for ≥10 s; time below threshold in minutes/night | SpO₂ measured via reflectance PPG on wrist device; nightly quality filter applied (epochs with >20% artifact rejected). Desaturation frequency = count of qualifying events per night. Daytime SpO₂ measured during active bouts defined above. |
| **Voice Biomarkers** | Pause duration, F0, MFCC coefficients, shimmer, jitter, speech rate, spectral features | Weekly audio recording (single session, patient counts 1–30 in preferred language) | Per-recording extraction; 7-day value = most recent available recording; trend = current minus prior week value | Audio processed via custom Python pipeline (librosa, praat-parselmouth). Pause duration = silent segments >200 ms. F0 estimated via autocorrelation (Praat). MFCCs (13 coefficients) computed on 25-ms frames, 10-ms step. Shimmer = mean absolute log ratio of successive amplitude peaks. Jitter = mean absolute deviation of successive F0 periods divided by mean F0 period. Speech rate = estimated syllable count / phonation time. |
| **Clinical Variables** | Medication class, NYHA class, LVEF category, NT-proBNP, prior hospitalization | Baseline and most recent clinic visit | Binary flags for medication classes; ordinal encoding for NYHA; log-transformation for NT-proBNP | Medication data extracted from electronic health record at enrollment. Beta-blocker: any beta-adrenergic blocking agent. SGLT2i: any sodium-glucose cotransporter-2 inhibitor. Diuretic dose standardized to furosemide equivalents (mg/day). LVEF category: HFrEF (<40%), HFmrEF (40–49%), HFpEF (≥50%). NT-proBNP log-transformed (natural log) prior to inclusion. |
| **Patient-Reported Symptoms** | Daily dyspnea, fatigue, edema reports | 7-day preceding KCCQ assessment | Mean dyspnea score (0–10), mean fatigue score (0–10), proportion of days with edema reported | Captured daily via BiovitalsHF® mobile app push notifications. Dyspnea: 'How short of breath are you today?' (0 = none, 10 = worst imaginable). Fatigue: 'How tired/fatigued are you today?' (0–10). Edema: binary yes/no. Non-response treated as missing; days with ≥4 of 7 responses required for inclusion of weekly summary. |

AEE = activity energy expenditure; FFT = fast Fourier transform; HFpEF = heart failure with preserved ejection fraction; HFrEF = heart failure with reduced ejection fraction; IS = interdaily stability; IV = intradaily variability; L5 = least active 5-hour mean; LVEF = left ventricular ejection fraction; M10 = most active 10-hour mean; MFCC = Mel-frequency cepstral coefficient; MET = metabolic equivalent of task; NT-proBNP = N-terminal pro-B-type natriuretic peptide; OLS = ordinary least squares; PPG = photoplethysmography; RA = relative amplitude; RR = respiratory rate or R-to-R interval (context-dependent); SGLT2i = sodium-glucose cotransporter-2 inhibitor.

# Supplementary Figure S1. Correlation matrix of top 20 LASSO-selected features

**Caption:** Pairwise Pearson correlation matrix among the top 20 features ranked by absolute LASSO coefficient magnitude. Features are ordered by coefficient magnitude along both axes (highest at top-left). Color scale: dark blue = strong negative correlation (r = −1.0); white = no correlation (r = 0); dark red = strong positive correlation (r = +1.0). Correlation computed on the training dataset (n = 115 observations) after z-score normalization. Cells are annotated with r values where |r| ≥ 0.40.

# Supplementary Figure S2. Individual patient KCCQ-12 trajectories: actual vs. predicted

**Caption:** Spaghetti plots of actual (observed) KCCQ-12 overall summary scores versus model-predicted scores across the 8-week study for individual patients in the held-out test set (n = 49 observations from approximately 18 patients). Each panel subplot shows one patient; x-axis = study week (0–8); y-axis = KCCQ-12 score (0–100). Solid blue line = actual KCCQ-12. Dashed orange line = linear mixed-effects model prediction. Shaded region = ±1 SE of the predicted value. A summary inset shows the overall predicted-vs-actual scatter plot (r = 0.77, R² = 0.58) across all test observations. Reference lines at KCCQ-12 = 65 (median threshold used for binary classification) are shown in grey.
